# One Health Approach on Dog Bites: Demographic and Associated Socioeconomic Factors in Southern Brazil

**DOI:** 10.3390/tropicalmed8040189

**Published:** 2023-03-25

**Authors:** Caroline Constantino, Evelyn Cristine Da Silva, Danieli Muchalak Dos Santos, Igor Adolfo Dexheimer Paploski, Marcia Oliveira Lopes, Vivien Midori Morikawa, Alexander Welker Biondo

**Affiliations:** 1 Graduate College of Veterinary Science, Federal University of Paraná (UFPR), Curitiba 80035-050, PR, Brazil; 2 State Secretary of Health, Apucarana 86800-140, PR, Brazil; 3 Institute of Biotechnology, São Paulo State University (UNESP), Tecomarias Avenue, Botucatu 18607-440, SP, Brazil; 4 Department of Collective Health, Federal University of Paraná State, Curitiba 80060-240, PR, Brazil; 5 Department of Veterinary Population Medicine, University of Minnesota, St. Paul, MN 55108, USA; 6 City Secretary of Environment, Curitiba 80810-000, PR, Brazil; 7 Department of Veterinary Medicine, Federal University of Paraná, Curitiba 80035-050, PR, Brazil; 8 Department of Comparative Pathobiology, Purdue University, West Lafayette, IN 47907, USA

**Keywords:** dog bites, human rabies prophylaxis, spatial analysis, associated factors, low income

## Abstract

Despite being an important public health issue, particularly due to rabies, dog bites and associated risk factors have rarely been assessed by health services from a One Health perspective. Accordingly, the present study aimed to assess dog biting and associated demographic and socioeconomic risk factors in Curitiba, the eighth-largest Brazilian city with approximately 1.87 million people, based on the post-exposure prophylaxis (PEP) rabies reports between January/2010 and December/2015. The total of 45,392 PEP reports corresponded to an average annual incidence of 4.17/1000 habitants, mainly affecting white (79.9%, 4.38/1000 population), males (53.1%, 4.81/1000 population), and children aged 0–9 years (20.1%, 6.9/1000 population), with severe accidents associated with older victims (*p* < 0.001) and mainly caused by dogs known to the victims. An increase of USD 100.00 in the median neighborhood income was associated with a 4.9% (95% CI: 3.8–6.1; *p* < 0.001) reduction in dog bites. In summary, dog biting occurrence was associated with victims’ low income, gender, race/color, and age; severe accidents were associated with elderly victims. As dog bites have been described as multifactorial events involving human, animal, and environmental factors, the characteristics presented herein should be used as a basis to define mitigation, control, and prevention strategies from a One Health perspective.

## 1. Introduction

Dog biting has been considered a characteristic natural behavior towards dominance, competition, or defense, leading to public health concerns such as dog-to-human aggression [1,2], requiring a One Health approach due to their multifactorial cause and impacts on human and animal health and welfare [3,4,5]. In addition to body wounds, dogs have been considered as the main rabies transmitters in urban settings, particularly in developing countries [6,7]. The risk of rabies transmission by a dog bite may be worsened by close human–dog contact and the high human-density population in major cities [2,8]. In such a scenario, urban settings of Brazil may be highly exposed, with the presence of dogs surpassing children in almost half of Brazilian households (46.1%), with the Southern region being above the nationwide average (57.4%) [9]. Based on the estimated dog population [6], less than 70% of Brazilian cities reach the target of 80% rabies vaccination.

Dog bites may directly affect health services due to hospitalizations, reconstructive surgeries, treatment of secondary bacterial infections, and psychological traumas, along with the costs of human rabies prophylaxis [2]. Additionally, dog-biting accidents may impact occupational health as a consequence of labor incapacity and worker absence, causing public and private service suspensions or delays such as postal and delivery services, garbage collection, water, electricity, and gas meter readings [10].

Despite the public health issues worldwide, and particularly in the Brazilian unified health system (Sistema Único de Saúde (SUS)), no study has focused on dog biting evaluation as the basis for the One Health approach and assessment [11]. Even so, a Brazilian nationwide online-based report has been mandatory for all medical attendance resulting from animal biting or scratching accidents, historically due to potential rabies transmission, as part of the post-exposure prophylaxis (PEP) rabies report [12]. Moreover, PEP has reportedly been the most frequent health event in several major cities [13], with dogs accounting for 81.7% of the overall 7.5 million PEP reports in Brazil between 2007 and 2019 [6].

Although analysis of dog biting reports may provide an important feedback tool for health services and professionals, such information has been limited to few studies focusing on rabies monitoring and prevention, along with the local demands of dog anti-rabies vaccination. Accordingly, based on the human PEP reports, the present study aims to assess dog bite accidents and associated demographic and socioeconomic risk factors in Curitiba, Southern Brazil.

## 2. Materials and Methods

Curitiba (25°25′40″ S and 49°16′23″ W), the capital of Parana State, was ranked as the eighth biggest Brazilian city with approximately 1.87 million inhabitants, classified as the only urban area located in a subtropical highland climate region. At the time of the survey, Curitiba presented a very high human development index (0.823), a 0.41 Gini Index [14], and 75 neighborhoods grouped into ten administrative regions, each with its own health surveillance sections called sanitary districts [15].

The occurrence of dog biting was obtained from PEP reports submitted between 1 January 2010 and 31 December 2015, publicly available in the Brazilian Information System for Notifiable Diseases (Sistema de Informação de Agravos de Notificação (SINAN)), an online database from SUS. Only PEP reports caused by dogs were gathered and analyzed when both the reporting health service and the victim were from Curitiba. As the last comprehensive Brazilian census occurred in 2010, the 2010–2015 period was chosen for the reliability of applied population data.

Only 14 of the 60 variables were obtained from SINAN medical PEP records, tabulated, and analyzed according to the event characteristics, including person bitten (age, gender, race/color, education), victim’s neighborhood residence, notification date, municipality, reporting health service, and accident-specific characteristics. Specific data collected on the accident included the type of rabies exposure (bites, scratches, licks, indirect contact, or other), the number, site, and depth of wounds, animal aggressor species (as inclusion criteria), and the possibility of dog monitoring. The data were described using absolute and relative frequencies. Statistical analyses were performed using commercially available software (Epi Info version 7.1.5.2, CDC, Atlanta, GA, USA and Stata version 17, StataCorp., 2021, Stata Statistical Software: Release 17. StataCorp LLC., College Station, TX, USA).

Bites were classified as mild when they resulted in superficial wounds, restricted to a few body areas and usually single trunk and limb wounds, or due to superficial skin wound biting, scratching, or licking. Accidents were classified as severe when wounds were located on the head, face, neck, hands, or soles of the feet or when deep, multiple, or extensive wounds in any body site were caused by dog nails or the licking of mucosa/broken skin. This classification, considering the type of rabies exposure, wound characteristics, and animal aggressor condition, was also used by the Brazilian Ministry of Health for human rabies prophylaxis management [12]. The frequency of dog bites that were classified as severe within different groups was compared using a Chi-Square test at the level of 5%.

Maps describing the average annual incidence of dog bites per neighborhood (Table S1 [Supplementary Materials]) were constructed using QGIS (Quantum Geographic Information System, version 2.16.1, Open-Source Geospatial Foundation Project). The median income of each neighborhood [16] (Table S3) and the population density per km^2^ [17] (Table S2) were illustrated to allow a spatial understanding of their relationship with the average annual incidence of dog bites. The victim’s address was assumed as the location where the accident occurred because this location is not a variable present in the PEP reports. Only reports monitoring dog aggressors were considered for analysis to minimize the error likelihood.

The association of the total number of dog bites per neighborhood (accounting for different exposure levels due to neighborhood population size) to the median income in dollars (average rate from 2010 to 2015) of the neighborhood was investigated using a negative binomial regression due to overdispersion of the count of dog bites in Stata version 17. The median neighborhood income was transformed to a scale of a hundred (100 = 1, 1000 = 10 and so on) to facilitate the observed effect on a more meaningful scale.

This study was approved by the Ethics Committee of the Curitiba City Secretary of Health (protocol code 62/2016).

Due to the Ethics Committee and current Brazilian laws on personal information and privacy, the original and complete database that supports the demographic findings of this study may be available only upon request to the corresponding author. Everything else, including average data and statistical analysis, are provided here in the Supplementary Materials (Tables S1–S3 and Figures S1,S2).

## 3. Results

From the PEP reports obtained during the period, 45,392/58,338 (77.8%) were due to dog bites, with victims living at Curitiba addresses. The average of dog biting per year was 7565 PEP, corresponding to a 4.17 annual average incidence of dog bites per thousand habitants, ranging from 3.98 in 2015 to 4.27 per thousand in 2014. Bites were mostly caused by dogs known to the victims (35,755/45,392; 78.8%), with most accidents reported in January (4399/45,392, 9.7%) and December (4210/45,392, 9.3%).

A total of 24,089/45,392 (53.1%) victims were male (4.81 per 1000 population), 9115/45,392 (20.1%) were aged between 0 and 9 years (6.91 per 1000 population), and 36,286/45,392 (79.9%) were white (4.38 per 1000 population), considered the most affected strata according to average annual incidence (Table 1). As 13,969/45,392 (30.8%) PEP report forms contained ignored or blank data, education level was not considered for analysis due to the unreliable database.

**Figure 1:**
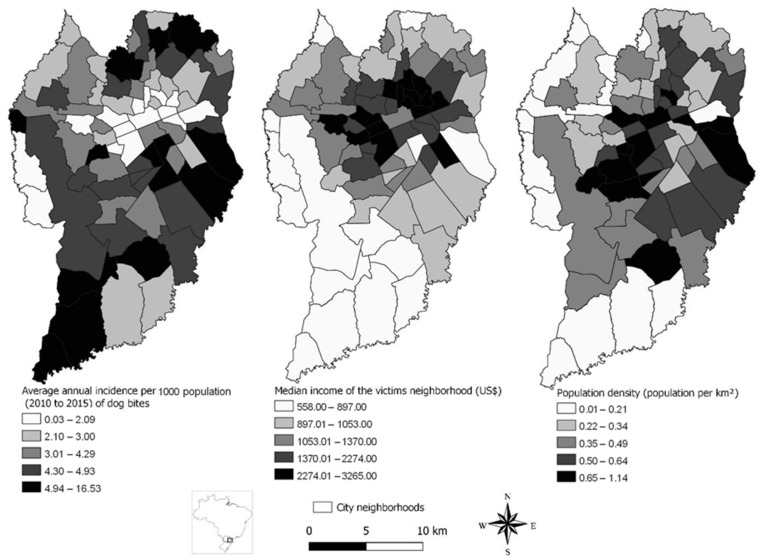
Geographic map of Curitiba city, Paraná State, Southern Brazil. Enlarged Curitiba maps show the spatial distribution of the average annual incidence of dog bites by victims’ neighborhood, the median income of the victims’ neighborhood, and human population density by neighborhood per km^2^ from 1 January 2010 to 31 December 2015 in Curitiba, Parana State, Southern Brazil.

Of the dog bites, 16,769/45,392 (36.9%) cases occurred in the lower limbs, with 23,196 (52.2%) multiple and 21,050 (46.4%) deep wounds. A total of 36,039/45,392 (79.4%) victims presented characteristics of a severe biting event. Severe accidents occurred more frequently with increasing age (*p* < 0.001), while the severity of the biting event was not statistically associated with gender (*p* = 0.308) (Figure S1). An increase of USD 100.00 in the neighborhood median income was associated with a 4.9% (95% CI: 3.8–6.1; *p* < 0.001) reduction in the number of dog bites after adjusting for human population size (Figure S2). The spatial distribution of the average annual incidence of dog bites per neighborhood, the median income of each neighborhood, and population density were presented (Figure 1).

## 4. Discussion

Dog biting has reportedly posed a public health burden worldwide and is considered a neglected problem for poor and other vulnerable populations [11]. The main associated concern has been human rabies transmission which has killed more than 55,000 people annually worldwide despite it being fully preventable by massive vaccination [7]. In Brazil, even in rabies-vaccination-controlled areas, dog bites still cause a great economic and health impact, given the costs of human rabies prophylaxis and treatment of secondary injuries, which may require resources that could be directed to supply other primary health demands [2,3,18].

Characterizing the human population frequently bitten by dogs and understanding the associated risk factors may help establish preventive actions and reduce their occurrence [2,19,20]. This study presents a valuable contribution to the field of public health, particularly concerning dog bites and associated risk factors from a One Health perspective. The research was conducted in Curitiba, the eighth-largest Brazilian city, and analyzed post-exposure prophylaxis (PEP) rabies reports between January/2010 and December/2015. The results revealed that dog biting was most common in white males and children aged 0–9 years; severe accidents were associated with older victims. The present study also identified socioeconomic-associated risk factors for dog bite occurrence. The study outcomes suggest that specific demographic groups were more likely bitten by dogs and had severe accidents and that the incidence distribution was not homogeneous in space, being associated with lower income. Such findings may provide substantial evidence for administrative agencies to better allocate resources, aiming to control and prevent dog-transmitted rabies, among other disorders, caused by unhealthy dog-human interaction [4,19].

The dog biting incidence observed herein was higher than found in other regions such as Campinas with 2.42 [21] and Minas Gerais State with 1.22 [13], both in Southeastern Brazil and in the USA with 1.1 per thousand habitants [2]. These statistics have accumulated the intrinsic differences among health service skills to identify dog-to-human aggression, the problems with access to health services and have also highlighted that different human populations might interact differently with dogs known to them [11,19], reflecting on the incidence of dog biting in a given population. Thus, predisposing and determinant factors in each One Health context should define policies for successful dog biting mitigation and prevention.

The frequency and severity of dog bites in several studies were previously associated with male and infant victims; males were more likely to be bitten due to their tendency of reckless behavior and more intimidating variation [22]. A study conducted in São Paulo State, Brazil, showed that the presence of a child in a house doubled the likelihood of dog bite incidents, while the presence of adults was considered a protective factor, decreasing the chances of accidents by 34% for each adult present [23]. Importantly, children were also more vulnerable to dog biting, mostly due to curious behavior and deficient self-defense, leading to an attempted escape and resulting in a higher likelihood of head and neck wounds, in particular as a result of their low height [2,13,24]. Additionally, children may mistakenly feel more comfortable with dogs they know, taking risky actions, allowing challenging proximity and unkind close contact [20]. Boys demonstrate touchier and girls more cautious behavior during dog–human interactions [1,24].

The present study has shown the highest dog biting incidence in males and children aged 0 to 9 years (Table 1), as previously described in Rio Grande do Sul [18] and Minas Gerais states [13], Brazil and Spain [19]. Surprisingly, reported severe accidents were more frequently associated with increasing age (*p* < 0.001) and not with human gender (*p* = 0.308), which may be related to the classification of mild and severe accidents [12] as elderly persons were mostly bitten by dogs in lower limbs, hands, and feet [19,25]; the same was observed in our study. Additionally, the elderly may present biting wound patterns caused by dogs either known and/or familiar to them [1].

Demographic factors and population socioeconomic conditions influence health or illness rates [26,27]. We found that an increase of USD 100.00 in a neighborhood’s median income was associated with a 4.9% (95% CI: 3.8–6.1; *p* < 0.001) lower incidence rate of dog bites. Such findings may indicate that low income and local development may also mirror local animal health with a lack of or insufficient basic veterinary services, including rabies vaccination, dog neutering/spaying, and responsible guardianship [11,28], particularly in vulnerable communities. A survey conducted by our research group in Curitiba showed an inverse correlation between neighborhood income and dog biting amongst local postmen [10], reflecting that households with a better infrastructure reduced the overall occupational exposure to dog bites.

Dog bites have also been associated with social and environmental conditions in Brazil [8,10] and Canada [29], indicating that local levels of human violence [4,29] and household structure may raise the likelihood of human–dog interactions [10], predisposing to dog bites. As vulnerable areas develop a higher human and animal overlapping populations, potential exposure to dog biting may also increase [8,10]. Interestingly, in our study, the spatial distribution of the average annual incidence of dog bites was not associated with a high human population density in Curitiba (Figure 1). Whether dog biting may be reduced by urban verticalization, mostly in downtown neighborhoods and concentrating the highest population per km^2^ but not the highest incidence of dog accidents at the time, should be further investigated.

The present study has some limitations, such as the reliance on PEP reports, which may not accurately reflect the true incidence of dog bites, as some people may not seek medical attention. Additionally, this study was not able to detect the breed of the dogs involved in biting incidents, which could have provided further insights into the risk factors for dog bites, as well as information concerning the animals’ size, age, gender, reproductive status and the circumstances of the accident, none of which were included in the PEP report at the time. Dog biting has been considered an instinctive animal behavior motivated by dominance, competition, or defense; in addition, the knowledge of animal characteristics surrounding aggressions might be used to define preventive strategies. In Brazil, dog bites were mostly caused by intact males [1,3,23,25] and young [30] dogs, domiciled and with no history of annual vaccination against rabies [31].

Although certain dog breeds have been reported to show a higher attack tendency, such as mixed-breed dogs, sheepdogs, and terriers [1,19,32], data should be analyzed cautiously as some breeds may be more popular in some regions, biasing the dog biting statistics [32]. In addition, the aggressor breed may not be faithfully recorded, and mixed-breed dogs are often described as purebred dogs [1].

Related to the animal characteristics, the size can also significantly influence the severity of the injuries, with medium and large animals causing the most severe injuries [1,5,23]. On the other hand, smaller dogs are more frequently involved in episodes of biting accidents [24]. Thus, further investigation of animal aggressor characteristics should be conducted to fully establish animal factors associated with dog biting, particularly in higher occurrence and/or socially vulnerable areas.

Although the study had access to a large number of reports, the secondary databases from health services used here showed other limitations, including the inaccuracy of identifying the geographical location in which the dog biting occurred, as this information is not included in the PEP report. As previous studies have suggested that victims were mostly bitten by their own dogs [13,19,20,23,25], in the vicinities of their own household [1,2], authors believe that victims herein may have also lived nearby the location where the dog biting occurred. As analyses were made only with PEP reports in which the aggressor dog was monitorable, dogs were more likely known and lived probably close to the victim.

As another limitation, PEP reports may be intrinsically biased by more severe accidents, as mild dog bites may require less medical care and thus generate less medical attendance and PEP reports. As such, the data reported herein may have captured a higher fraction of the total number of severe biting events and underestimated the number of non-severe dog bites. Additionally, specific demographic groups, such as children, may be more likely to seek or be taken to medical care [2,22]. Another potential bias source may be associated with access to health services; certain populations within a city may receive different disinformation about access and show refusal of health resources [28], which may also lead to biased demographic or geographical reporting of dog bites [33,34]. As a consequence of the above factors, the number and incidence of dog biting herein may be even higher; previous studies indicated a three-fold underestimation between official reports and actual cases [22].

Despite these limitations, dog bites herein have been shown to be a multifactorial event, intrinsically and statistically correlated with demographic and socioeconomic human, dog, and environmental characteristics. Such a scenario demands a One Health approach in mitigating and preventive measures, aimed at reducing the number of dog bites and establishing preventive measures on the three health-related components. Until now, the most effective actions to reduce dog biting have included responsible dog guardianship, dog registration and identification, enforcement laws, dog neutering/spaying, training, and knowledge of natural dog behavior [2,35]. Given the findings of the present study, actions prioritizing the most socially and economically vulnerable human areas should be encouraged by decision-makers and governments, as low-income neighborhoods have been related to a higher incidence of dog bites and, consequently, more exposure to autochthonal dog rabies transmission and spread in endemic countries. Finally, fighting against social inequality should be the central goal of public policies, as it statistically decreases dog bites and improves population health [27,36].

Overall, the findings herein have important implications for public health policy and practice, particularly in relation to the prevention and control of dog bites. By considering the socioeconomic factors and adopting a One Health approach, public health agencies can design effective strategies to reduce the incidence of dog bites and improve public health outcomes.

## 5. Conclusions

In summary, dog bite occurrence was associated with low income, gender, race/color, and age of victims and involved known dogs; in addition, severe accidents were associated with older victims. One of the key findings was the association between dog biting and the victims’ socioeconomic status, indicating that an increase of USD 100.00 in the median neighborhood income was associated with a 4.9% reduction in dog biting, highlighting the importance of considering socioeconomic factors when developing prevention and control strategies for dog bites. Furthermore, the study has shed light on the multifactorial nature of dog biting, emphasizing the need for a One Health perspective. As dog bites involve human, animal, and environmental factors, the presented characteristics should be used as a basis for defining mitigation, control, and prevention strategies, prioritizing the most affected low-income populations and vulnerable areas.

## Figures and Tables

**Table 1:** Distribution of the variables and incidence per 1000 habitants related to gender, age, and race/color of dog bite victims in Curitiba based on PEP reports, Southern Brazil, between 1 January 2010 and 31 December 2015.

Variables	Dog Bites n (%)	Population (IBGE, 2010)	Cumulative Incidence per 1000 Population	Average Annual Incidence per 1000 Population
Gender				
Male	24,089 (53.1)	835,115	28.85	4.81
Female	21,298 (46.9)	916,792	23.23	3.87
Age (years)				
0–9	9115 (20.1)	219,967	41.4	6.91
10–19	7530 (16.6)	269,505	27.9	4.66
20–29	6173 (13.6)	324,304	19.0	3.17
30–39	5743 (12.7)	2,932,233	19.6	3.26
40–49	5581 (12.3)	253,068	22.1	3.68
50–59	5238 (11.5)	193,741	27.0	4.51
60–69	3466 (7.6)	111,753	31.0	5.17
70–79	1867 (4.1)	59,092	31.6	5.27
≥80 years	679 (1.5)	27,244	24.9	4.15
Race/color				
White	36,286 (79.9)	1,380,012	26.29	4.38
Black	1058 (2.3)	49,320	21.45	3.58
Yellow	446 (1.0)	23,888	18.67	3.11
Indigenous	3010 (6.6)	296,140	10.16	1.69
Ignored or blank	44 (0.1)	2421	18.17	3.03

## Data Availability

Due to the Ethics Committee and current Brazilian laws on personal information and privacy, the original and complete database that supports the demographic find-ings of this study may be available only upon request to the corresponding author. Everything else, including average data and statistical analysis, are provided here in the Supplementary Materials.

## Supplementary Materials

The following supporting information can be downloaded at: https://www.mdpi.com/article/10.3390/tropicalmed8040189/s1, Table S1: Data of annual dog bites incidence by neighborhoods of Curitiba, Southern Brazil, between 1 January 2010 and 31 December 2015; Table S2: Data of human density by neighborhoods of Curitiba, Southern Brazil, 2010; Table S3: Data of the median income by neighborhoods of Curitiba, Southern Brazil, 2010; Figure S1: A Chi-Square test of age and severe accidents; and Figure S2: Negative binominal regression test).
