# Supplementary material for: One Health Approach on Dog Bites: Demographic and Associated Socioeconomic Factors in Southern Brazil
Source: Trop Med Infect Dis. 2023 Mar 25;8(4):189. doi: 10.3390/tropicalmed8040189 (PMC10146334; doi:10.3390/tropicalmed8040189)
Supplement: Supplementary file 1 [file tropicalmed-08-00189-s001.zip › Figure S1.pdf]

Expandir menu | Reduzir menu

- Início
- Informação e ajuda
- Idioma/Opções/Configurações
- Calculadora
- Cálculos
  - Taxa de Mort.Pad.
  - Proporção
  - Tabela 2 x 2
  - Dose-Resposta
  - Tabela L x C
  - Casos-Controle pareados
  - Triagem
- Pessoas-Tempo
  - 1 taxa
  - Comparar 2 taxas
- Variáveis contínuas
  - Média IC
  - Mediana/%il IC
  - Teste t
  - ANOVA
- Tamanho da amostra
- Poder
- Números aleatórios
- Buscas
  - Google--Internet
  - PubMed--MEDLARS
- Links na Internet
- Baixar OpenEpi
- Desenvolvimento

Início
Entrar dados
Resultados
Exemplos
Ajuda

Análise de tabela simples

	Var 2			
	85	356	0	441
Var 1	1131	3360	6	4497
	1346	3876	9	5231
	930	3045	6	3981
	689	2405	4	3098
	674	2439	5	3118
	644	2377	6	3027
	546	2454	7	3007
	512	2226	6	2744
	505	2240	2	2747
	474	2400	1	2875
	471	2201	1	2673
	418	1944	3	2365
	296	1572	2	1870
	206	1216	1	1423
	169	862	2	1033
	102	602	0	704
	50	326	1	377
	15	127	0	142
	6	33	0	39
	9269	36061	62	45392

Qui-quadrado para Tabela L x C

Qui-quadrado	373.5
Graus de liberdade	38
Valor-p	<0.0000001

Cochran recomenda aceitar o qui-quadrado se:
1. Menos de 20% das células tenham valor esperado <5.
2. Nenhuma célula tem um valor esperado <1.

Nesta tabela:
28% of 60 cells have expected values < 5.
5 cells have expected values < 1.

Esta tabela NÃO CUMPRE os critérios de Cochran.

Valor esperado= total de linhas * total de colunas/total

Rosner, B. Fundamentos de Bioestatística. 5a ed. Duxbury Thompson Learning 2000; p. 395

Resultados do OpenEpi, Versão 3, calculadora de código aberto--RbyC
Imprima a partir do navegador com ctrl-P
ou selecione o texto para copiar e colar em outros programas.

Parece que não há nenhum exemplo para este exercício.
