# Supplementary material for: One Health Approach on Dog Bites: Demographic and Associated Socioeconomic Factors in Southern Brazil
Source: Trop Med Infect Dis. 2023 Mar 25;8(4):189. doi: 10.3390/tropicalmed8040189 (PMC10146334; doi:10.3390/tropicalmed8040189)
Supplement: Supplementary file 1 [file tropicalmed-08-00189-s001.zip › Figure S2.pdf]

```
. nbreg n_mordeduras rendamediadolares_cem, dispersion(mean) exposure(num_habitantes) irr

Fitting Poisson model:

Iteration 0:   log likelihood =  -1501.905
Iteration 1:   log likelihood = -1501.7905
Iteration 2:   log likelihood = -1501.7905

Fitting constant-only model:

Iteration 0:   log likelihood = -508.82329
Iteration 1:   log likelihood = -503.05528
Iteration 2:   log likelihood = -489.13435
Iteration 3:   log likelihood = -488.93696
Iteration 4:   log likelihood = -488.93622
Iteration 5:   log likelihood = -488.93622

Fitting full model:

Iteration 0:   log likelihood = -473.22552
Iteration 1:   log likelihood = -469.19211
Iteration 2:   log likelihood = -467.63033
Iteration 3:   log likelihood = -467.62291
Iteration 4:   log likelihood = -467.62291

Negative binomial regression                    Number of obs     =         75
                                                LR chi2(1)        =      42.63
Dispersion     = mean                           Prob > chi2       =     0.0000
Log likelihood = -467.62291                     Pseudo R2         =     0.0436

------------------------------------------------------------------------------------------
         n_mordeduras |        IRR   Std. Err.      z    P>|z|     [95% Conf. Interval]
----------------------+-------------------------------------------------------------------
rendamediadolares_cem |   .9505122   .0060369    -7.99   0.000     .9387534    .9624183
                _cons |    .047888   .0053081   -27.42   0.000     .0385367    .0595083
  ln(num_habitantes)  |          1  (exposure)
----------------------+-------------------------------------------------------------------
             /lnalpha |   -1.77875   .1803606                     -2.13225   -1.425249
----------------------+-------------------------------------------------------------------
                alpha |   .1688491   .0304537                      .1185702    .2404485
------------------------------------------------------------------------------------------
Note: Estimates are transformed only in the first equation.
Note: _cons estimates baseline incidence rate.
LR test of alpha=0: chibar2(01) = 2068.34             Prob >= chibar2 = 0.000
```
