# Supplementary material for: One Health Approach on Dog Bites: Demographic and Associated Socioeconomic Factors in Southern Brazil
Source: Trop Med Infect Dis. 2023 Mar 25;8(4):189. doi: 10.3390/tropicalmed8040189 (PMC10146334; doi:10.3390/tropicalmed8040189)
Supplement: Supplementary file 1 [file tropicalmed-08-00189-s001.zip › Table S1.pdf]

Table S1: Data of annual dog bites incidence by neighborhood of Curitiba, Southern Brazil, between January 1st 2010 and December 31st 2015.

Neighborhood number	Neighborhood code	Neighborhood name	Cumulative incidence/1,000 pop.	Average annual incidence/1,000 pop.
1	4106902050	Abranches	30.93	5.16
2	4106902009	Agua Verde	7.99	1.33
3	4106902014	Ahu	13.73	2.29
4	4106902064	Alto Boqueirao	26.72	4.45
5	4106902004	Alto da Gloria	12.8	2.13
6	4106902005	Alto da XV	9.85	1.64
7	4106902055	Atuba	20.9	3.48
8	4106902068	Augusta	6.67	1.11
9	4106902035	Bacacheri	17.99	3
10	4106902036	Bairro Alto	29.93	4.99
11	4106902052	Barreirinha	34.3	5.72
12	4106902010	Batel	6.8	1.13
13	4106902011	Bigorrilho	6.71	1.12
14	4106902034	Boa Vista	27.34	4.56
15	4106902013	Bom Retiro	17.65	2.94
16	4106902056	Boqueirao	30.32	5.05
17	4106902061	Butiatuvinha	18.41	3.07
18	4106902016	Cabral	13.02	2.17
19	4106902051	Cachoeira	18.9	3.15
20	4106902021	Cajuru	37.7	6.28
21	4106902030	Campina do Siqueira	20.61	3.44
22	4106902044	Campo Comprido	19.78	3.3
23	4106902071	Campo do Santana	37.96	6.33
24	4106902020	Capao da Imbuia	28.87	4.81
25	4106902058	Capao Raso	24.76	4.13
26	4106902047	Cascatinha	13.42	2.24
27	4106902070	Caximba	42.43	7.07

28	4106902001	Centro	12.79	2.13
29	4106902003	Centro Cívico	10.45	1.74
30	4106902075	CIC	28.06	4.68
31	4106902006	Cristo Rei	8.41	1.4
32	4106902039	Fanny	29.83	4.97
33	4106902042	Fazendinha	27.18	4.53
34	4106902072	Ganchinho	18.34	3.06
35	4106902023	Guabirotuba	21.73	3.62
36	4106902026	Guaíra	27.84	4.64
37	4106902038	Hauer	36.12	6.02
38	4106902017	Hugo Lange	7.96	1.33
39	4106902007	Jardim Botânico	18.15	3.02
40	4106902022	Jardim das Americas	15.67	2.61
41	4106902018	Jardim Social	13.34	2.22
42	4106902015	Juveve	8.38	1.4
43	4106902062	Lamenha Pequena	18.94	3.16
44	4106902040	Lindoia	28.54	4.76
45	4106902012	Merces	13.79	2.3
46	4106902045	Mossungue	13.66	2.28
47	4106902041	Novo Mundo	28.48	4.75
48	4106902059	Orleans	24.06	4.01
49	4106902025	Parolin	44.66	7.44
50	4106902032	Pilarzinho	31.67	5.28
51	4106902066	Pinheirinho	29.74	4.96
52	4106902027	Portao	18.47	3.08
53	4106902024	Prado Velho	61.05	10.17
54	4106902008	Reboucas	20.75	3.46
55	4106902069	Riviera	110.73	18.45
56	4106902053	Santa Candida	37.09	6.18
57	4106902063	Santa Felicidade	27.24	4.54
58	4106902043	Santa Quiteria	34.7	5.78
59	4106902046	Santo Inacio	22.33	3.72

60	4106902060	Sao Braz	28.14	4.69
61	4106902002	Sao Francisco	12.07	2.01
62	4106902048	Sao Joao	15.99	2.66
63	4106902033	Sao Lourenco	19.12	3.19
64	4106902067	Sao Miguel	0.21	0.03
65	4106902029	Seminario	12.84	2.14
66	4106902065	Sítio Cercado	32.22	5.37
67	4106902049	Taboao	20.91	3.48
68	4106902019	Taruma	11.4	1.9
69	4106902074	Tatuquara	36.43	6.07
70	4106902054	Tingui	28.57	4.76
71	4106902037	Uberaba	32.38	5.4
72	4106902073	Umbara	15.64	2.61
73	4106902028	Vila Izabel	11.8	1.97
74	4106902031	Vista Alegre	28.31	4.72
75	4106902057	Xaxim	22.89	3.82
