# Supplementary material for: One Health Approach on Dog Bites: Demographic and Associated Socioeconomic Factors in Southern Brazil
Source: Trop Med Infect Dis. 2023 Mar 25;8(4):189. doi: 10.3390/tropicalmed8040189 (PMC10146334; doi:10.3390/tropicalmed8040189)
Supplement: Supplementary file 1 [file tropicalmed-08-00189-s001.zip › Table S2.pdf]

Table S2: Data of human density by neighborhood of Curitiba, Southern Brazil, 2010.

Neighborhood number	Neighborhood code	Neighborhood name	Population density per km²	Human density (popul./km²)
1	4106902050	Abranches	30.56	0.31
2	4106902009	Agua Verde	107.74	1.08
3	4106902014	Ahu	62.03	0.62
4	4106902064	Alto Boqueirao	44.77	0.45
5	4106902004	Alto da Gloria	63.37	0.63
6	4106902005	Alto da XV	57.43	0.57
7	4106902055	Atuba	38.31	0.38
8	4106902068	Augusta	7.19	0.07
9	4106902035	Bacacheri	33.67	0.34
10	4106902036	Bairro Alto	64.03	0.64
11	4106902052	Barreirinha	49.76	0.5
12	4106902010	Batel	62.6	0.63
13	4106902011	Bigorrilho	97.83	0.98
14	4106902034	Boa Vista	59.44	0.59
15	4106902013	Bom Retiro	27.16	0.27
16	4106902056	Boqueirao	49.63	0.5
17	4106902061	Butiatuvinha	12.33	0.12
18	4106902016	Cabral	64.44	0.64
19	4106902051	Cachoeira	28.91	0.29
20	4106902021	Cajuru	81.57	0.82
21	4106902030	Campina do Siqueira	43.16	0.43
22	4106902044	Campo Comprido	33.62	0.34
23	4106902071	Campo do Santana	12.35	0.12
24	4106902020	Capao da Imbuia	110.99	1.11
25	4106902058	Capao Raso	40.54	0.41
26	4106902047	Cascatinha	9.9	0.1
27	4106902070	Caximba	3.07	0.03
28	4106902001	Centro	113.56	1.14
29	4106902003	Centro Cǒvico	50.5	0.51

30	4106902075	CIC	40.3	0.4
31	4106902006	Cristo Rei	95.34	0.95
32	4106902039	Fanny	41.69	0.42
33	4106902042	Fazendinha	75.39	0.75
34	4106902072	Ganchinho	9.84	0.1
35	4106902023	Guabirotuba	43.18	0.43
36	4106902026	Guaðra	64.48	0.64
37	4106902038	Hauer	33.37	0.33
38	4106902017	Hugo Lange	28.64	0.29
39	4106902007	Jardim Bot?nico	22.39	0.22
40	4106902022	Jardim das Americas	39.68	0.4
41	4106902018	Jardim Social	29.09	0.29
42	4106902015	Juveve	94.0	0.94
43	4106902062	Lamenha Pequena	3.14	0.03
44	4106902040	Lindoia	72.64	0.73
45	4106902012	Merces	35.92	0.36
46	4106902045	Mossungue	28.72	0.29
47	4106902041	Novo Mundo	74.07	0.74
48	4106902059	Orleans	15.82	0.16
49	4106902025	Parolin	51.16	0.51
50	4106902032	Pilarzinho	40.09	0.4
51	4106902066	Pinheirinho	47.94	0.48
52	4106902027	Portao	72.82	0.73
53	4106902024	Prado Velho	24.97	0.25
54	4106902008	Reboucas	50.19	0.5
55	4106902069	Riviera	1.19	0.01
56	4106902053	Santa Candida	32.19	0.32
57	4106902063	Santa Felicidade	25.93	0.26
58	4106902043	Santa Quiteria	57.39	0.57
59	4106902046	Santo Inacio	19.34	0.19
60	4106902060	Sao Braz	46.56	0.47
61	4106902002	Sao Francisco	45.26	0.45

62	4106902048	Sao Joao	10.72	0.11
63	4106902033	Sao Lourenco	27.59	0.28
64	4106902067	Sao Miguel	6.33	0.06
65	4106902029	Seminario	32.26	0.32
66	4106902065	Sðtio Cercado	103.15	1.03
67	4106902049	Taboao	18.58	0.19
68	4106902019	Taruma	19.36	0.19
69	4106902074	Tatuquara	46.98	0.47
70	4106902054	Tingui	57.26	0.57
71	4106902037	Uberaba	50.71	0.51
72	4106902073	Umbara	8.34	0.08
73	4106902028	Vila Izabel	95.43	0.95
74	4106902031	Vista Alegre	30.28	0.3
75	4106902057	Xaxim	62.94	0.63
