# Supplementary material for: One Health Approach on Dog Bites: Demographic and Associated Socioeconomic Factors in Southern Brazil
Source: Trop Med Infect Dis. 2023 Mar 25;8(4):189. doi: 10.3390/tropicalmed8040189 (PMC10146334; doi:10.3390/tropicalmed8040189)
Supplement: Supplementary file 1 [file tropicalmed-08-00189-s001.zip › Table S3.pdf]

Table S3: Data of the median income by neighborhood of Curitiba, Southern Brazil, 2010.

Neighborhood number	Neighborhood code	Neighborhood name	Median income of neighborhood (USD,2012)
1	4106902050	ABRANCHES	917.81
2	4106902009	ÁGUA VERDE	2283.11
3	4106902014	AHÚ	2374.43
4	4106902064	ALTO BOQUEIRÃO	913.24
5	4106902004	ALTO DA GLÓRIA	2374.43
6	4106902005	ALTO DA RUA XV	2283.11
7	4106902055	ATUBA	1091.32
8	4106902068	AUGUSTA	686.07
9	4106902035	BACACHERI	1917.81
10	4106902036	BAIRRO ALTO	1004.57
11	4106902052	BARREIRINHA	1050.23
12	4106902010	BATEL	3196.35
13	4106902011	BIGORRILHO	2700.91
14	4106902034	BOA VISTA	1369.86
15	4106902013	BOM RETIRO	2652.97
16	4106902056	BOQUEIRÃO	1004.57
17	4106902061	BUTIATUVINHA	913.24
18	4106902016	CABRAL	2652.97
19	4106902051	CACHOEIRA	753.42
20	4106902021	CAJURU	805.71
21	4106902030	CAMPINA DO SIQUEIRA	1826.48
22	4106902044	CAMPO COMPRIDO	1150.68
23	4106902071	CAMPO DE SANTANA	684.93
24	4106902020	CAPÃO DA IMBUIA	1141.55
25	4106902058	CAPÃO RASO	981.74
26	4106902047	CASCATINHA	1826.48
27	4106902070	CAXIMBA	577.63
28	4106902001	CENTRO	1369.86
29	4106902003	CENTRO CÍVICO	2283.11

30	4106902075	CIDADE INDUSTRIAL DE CURITIBA	796.8
31	4106902006	CRISTO REI	2271.69
32	4106902039	FANNY	1187.21
33	4106902042	FAZENDINHA	917.81
34	4106902072	GANCHINHO	643.84
35	4106902023	GUABIROTUBA	1598.17
36	4106902026	GUAÍRA	1054.79
37	4106902038	HAUER	1150.68
38	4106902017	HUGO LANGE	2735.16
39	4106902007	JARDIM BOTÂNICO	1506.85
40	4106902022	JARDIM DAS AMÉRICAS	2283.11
41	4106902018	JARDIM SOCIAL	3264.84
42	4106902015	JUVEVÊ	2511.42
43	4106902062	LAMENHA PEQUENA	732.88
44	4106902040	LINDÓIA	913.24
45	4106902012	MERCÊS	1826.48
46	4106902045	MOSSUNGUÊ	2739.73
47	4106902041	NOVO MUNDO	1141.55
48	4106902059	ORLEANS	1141.55
49	4106902025	PAROLIN	913.24
50	4106902032	PILARZINHO	1004.57
51	4106902066	PINHEIRINHO	831.05
52	4106902027	PORTÃO	1648.4
53	4106902024	PRADO VELHO	558.22
54	4106902008	REBOUÇAS	1552.51
55	4106902069	RIVIERA	684.93
56	4106902053	SANTA CÂNDIDA	913.24
57	4106902063	SANTA FELICIDADE	1324.2
58	4106902043	SANTA QUITÉRIA	1187.21
59	4106902046	SANTO INÁCIO	1369.86
60	4106902060	SÃO BRAZ	1095.89
61	4106902002	SÃO FRANCISCO	1598.17

62	4106902048	SÃO JOÃO	1004.57
63	4106902033	SÃO LOURENÇO	2146.12
64	4106902067	SÃO MIGUEL	639.27
65	4106902029	SEMINÁRIO	2330.59
66	4106902065	SÍTIO CERCADO	776.26
67	4106902049	TABOÃO	1089.04
68	4106902019	TARUMÃ	1917.81
69	4106902074	TATUQUARA	639.27
70	4106902054	TINGUI	1232.88
71	4106902037	UBERABA	913.24
72	4106902073	UMBARÁ	776.26
73	4106902028	VILA IZABEL	2009.13
74	4106902031	VISTA ALEGRE	1509.13
75	4106902057	XAXIM	958.9
